# Improved Evaluation Metrics for Sentence Suggestions in Nursing and Elderly Care Record Applications

**DOI:** 10.3390/healthcare12030367

**Published:** 2024-01-31

**Authors:** Defry Hamdhana, Haru Kaneko, John Noel Victorino, Sozo Inoue

**Affiliations:** 1 Graduate School of Life Science and Systems Engineering, Kyushu Institute of Technology, Kitakyushu 808-0196, Japansozo@brain.kyutech.ac.jp (S.I.); 2 Department of Informatics, Universitas Malikussaleh, Lhokseumawe 24355, Indonesia

**Keywords:** sentence suggestion, nursing care record, evaluation metrics

## Abstract

This paper presents a new approach called EmbedHDP, which aims to enhance the evaluation models utilized for assessing sentence suggestions in nursing care record applications. The primary objective is to determine the alignment of the proposed evaluation metric with human evaluators who are caregivers. It is crucial due to the direct relevance of the provided provided to the health or condition of the elderly. The motivation for this proposal arises from challenges observed in previous models. Our analysis examines the mechanisms of current evaluation metrics such as BERTScore, cosine similarity, ROUGE, and BLEU to achieve reliable metrics evaluation. Several limitations were identified. In some cases, BERTScore encountered difficulties in effectively evaluating the nursing care record domain and consistently providing quality assessments of generated sentence suggestions above 60%. Cosine similarity is a widely used method, but it has limitations regarding word order. This can lead to potential misjudgments of semantic differences within similar word sets. Another technique, ROUGE, relies on lexical overlap but tends to ignore semantic accuracy. Additionally, while BLEU is helpful, it may not fully capture semantic coherence in its evaluations. After calculating the correlation coefficient, it was found that EmbedHDP is effective in evaluating nurse care records due to its ability to handle a variety of sentence structures and medical terminology, providing differentiated and contextually relevant assessments. Additionally, this research used a dataset comprising 320 pairs of sentences with correspondingly equivalent lengths. The results revealed that EmbedHDP outperformed other evaluation models, achieving a coefficient score of 61%, followed by cosine similarity, with a score of 59%, and BERTScore, with 58%. This shows the effectiveness of our proposed approach in improving the evaluation of sentence suggestions in nursing care record applications.

## 1. Introduction

Nursing care records represent a compilation of electronic health records documenting the services and healthcare events provided to the elderly. Nursing care records include diagnoses, examination results, treatment plans, prescribed medications, and medical procedures undertaken [1]. These records precisely document the care provided by healthcare professionals, ranging from nurses/caregivers to physicians. Additionally, nursing care records incorporate details concerning hospital visits or engagements with other healthcare facilities. As comprehensive electronic documentation of healthcare interactions, nursing care records are pivotal in maintaining a detailed individual account of the health status and medical treatment the elderly receive.

Sentence suggestions are recommendations or predictions provided by a language model or text input system to assist users in constructing sentences effectively. The function of sentence suggestion in nursing care record applications is to complete a sentence based on the words inputted by the caregivers. Therefore, the model must understand the inputted word structure and predict the correct word for completion in a sentence [2,3]. An expected outcome of sentence suggestions is the completion of sentences similar to sentences previously used by the caregiver. The theoretical interest arising from the role of creating effective sentence suggestions makes a major contribution to a deeper understanding of the complex syntactic and semantic issues specific to nursing care notes. In nursing care records, we find short or incomplete sentence structures that are still relevant for further exploration. As a result, language modeling in the healthcare context is enhanced, improving communication and knowledge representation. On a practical level, the importance of sentence suggestions in care record applications is underscored by their potential to simplify and optimize the workflow of healthcare professionals. Providing accurate and contextually relevant sentence suggestions facilitates fast and precise data entry into the care record [4]. This significantly saves healthcare providers’ time and ensures the accuracy and completeness of medical documentation [5]. Furthermore, the practical application of well-crafted sentence suggestions in care notes promises to improve patient care outcomes significantly. It will be beneficial for healthcare professionals to improve overall healthcare efficiency.

The generated sentence suggestions must be based on the same information as the actual ground-truth sentences because nursing care records contain vital information about elders’ health and well-being. A challenge encountered in this study is the development of appropriate evaluation metrics for assessing the quality of sentence suggestions within nursing care records. Different sentence structures and specialized medical terminology pose challenges beyond the capabilities of conventional metrics designed to evaluate well-formed sentences and general language comprehension. Subjectivity in human evaluation, rooted in caregivers’ contextual understanding of elderly care [6], adds complexity, making it challenging to create automated metrics that authentically mimic human judgment. Additionally, the context sensitivity of nursing care records, where information is highly context-dependent [7], requires nuanced evaluation methods that existing metrics may struggle to provide. A further challenge arises because Japanese contains non-conventional linguistic structures, making using current evaluation metrics in nursing care record applications difficult. In other words, the unique structure and grammar of the language make generating and evaluating sentence suggestions in Japanese complex [8]. The non-standard architecture of Japanese sentence structures requires specialized adaptation in language models and evaluation metrics. Addressing these challenges is crucial to improving the accuracy of evaluation metrics tailored for the distinct characteristics of nursing care record sentences and content.

In conducting a thorough survey of the existing metrics, it becomes apparent that a comprehensive analysis may not be feasible due to constraints on resources and time. Consequently, a strategic decision was made to focus on critical metrics with significant weight and relevance. Our analysis examines the mechanisms of current evaluation metrics such as BERTScore, cosine similarity, ROUGE, and BLEU to achieve reliable metrics evaluation. Utilizing sample data assessed by three caregivers working in elderly care facilities in Japan, we identify certain shortcomings in current evaluation metrics when measuring similar information between sentence suggestions and the ground truth. BERTScore uses pre-trained BERT models that ineffectively capture domain-specific nuances such as nurse care records. Cosine similarity fails to consider word order, potentially causing errors in assessing semantic differences in groups of similar words. ROUGE tends to ignore semantic accuracy because it bases its evaluation on lexical overlap, ignoring context and word meaning. In addition, BLEU ignores semantic coherence. This exploration underscores the importance of refining existing evaluation metrics to effectively gauge the nuanced nature of sentence suggestions within the context of elderly care facilities.

In conclusion, we aim to solve the issue of evaluating the quality of sentence suggestions in nursing care records, which have the characteristics of diverse sentence structures and specialized medical terminology. We propose EmbedHDP, a novel hybrid topic model integrated with word embedding, intending to analyze the quality of sentence suggestion generation in nursing care record applications. Using hierarchical Dirichlet processes (HDPs) to train models of evaluation metrics for incomplete or fragmented nursing records is a sensible approach. The flexibility of HDPs, with no prior setting for the number of topics, makes them effective at handling non-standard sentence structures. In addition, their hierarchical structures enhance adaptability to complex and uncertain sentence structures. Meanwhile, word embedding in EmbedHDP is helpful for care records containing occasional medical terminology. EmbedHDP calculates topic distribution during model training using a dictionary containing words frequently used in care records, such as medical terms. In this study, we want to emphasize that Bag-of-Words (BoW) remains unable to deal with the context sensitivity of care records. Therefore, we propose a model that uses word embedding to create vectors that capture the semantic meanings of words. Through word embedding, it is possible to gain a deeper understanding of sentence context.

We used a dataset comprising 320 pairs of sentences with correspondingly equivalent lengths and evaluated them using both EmbedHDP and the current evaluation metrics. With caregiver assessments as a benchmark, we calculated the coefficient score for each evaluation model. The results indicate that our proposed evaluation model, EmbedHDP, outperformed other evaluation models, achieving a score of 61%, followed by cosine similarity, with 59%, and BERTScore, with 58%.

The contributions of this work are listed as follows:

1. We collected a dataset comprising 390 pairs of sentences, consisting of sentence suggestions and their corresponding ground truth. Three caregivers meticulously evaluated this dataset and utilized it as the benchmark ground truth.
2. We performed testing and analysis on current evaluation metrics such as BERTScore, cosine similarity, ROUGE, and BLEU to assess the quality of sentence suggestions in nursing care applications.
3. We introduced an innovative evaluation model featuring a novel approach that significantly improved the performance compared to existing evaluation models, aligning more closely with caregiver assessments.

These three contributions outlined above share a commonality rather than diverging from one another. They represent a comprehensive approach, including rigorous data collection, examination and analysis of current evaluation metrics, and the introduction of innovative new evaluation models. Together, these contributions form a cohesive and integrated effort to advance the evaluation of sentence suggestions in nursing care applications.

## 2. Related Works

In this chapter, we discuss several research studies conducted by relevant experts. These studies have inspired us to propose a new model for evaluating the quality of sentence suggestions, specifically for nursing care records. We also explore the existing evaluation metrics used to assess the quality of sentence suggestions.

### 2.1. Nursing Care Records

Nursing care records aim to record elders’ medical history, diagnoses, and treatment by doctors or other health professionals. Elderly care can be applied in nursing because it addresses the unique healthcare needs and considerations associated with the aging population, encompassing a holistic approach that involves physical, mental, and emotional well-being. Integrating elderly care into nursing care record applications ensures a personalized and comprehensive healthcare experience, considering the specific challenges and requirements of elderly individuals. This may involve features such as personalized health plans, medication management, mobility support, and monitoring of vital signs, all aimed at optimizing the quality of care provided to older adults. Using a time series approach, Caballero and Akella [9] developed a model to predict elders’ health conditions from nursing care applications. They underscored the importance of technology to increase understanding of elderly health status and enable more informed and effective decision making in elderly care.

Initially, nursing care records only consisted of digital medical documents that were easily searchable and accessible to health professionals. However, the development of nursing care records in health services refers to the electronic storage and sharing of elderly medical records in the healthcare system and has helped change the way the healthcare system works [10]. Some benefits of using nursing care records are increased efficiency and quality of health care, improve patient safety, and facilitation of research and development of drugs and treatments. However, with technological developments, nursing care records are now more complex and capable of collecting, processing, and analyzing patient health data on a large scale.

In this study, we used FonLog as a nursing care record application installed in more than 30 healthcare facilities in Japan. FonLog [11] is a mobile application designed as a data collection tool for human activity recognition in nursing services. Thus, caregivers can easily identify and record patient activities using a mobile phone, with key advantages such as recording the targeted patient, an easy-to-use interface, a recognition feedback interface, other customizable record details, instant activity, and offline accessibility. As a default, FonLog has 88 activity types in Japanese. Herein, we focus on providing sentence suggestions for special notes (特記事項) in 31 activity types and containing free format record text, as shown in Table 1.

**Figure 1:**
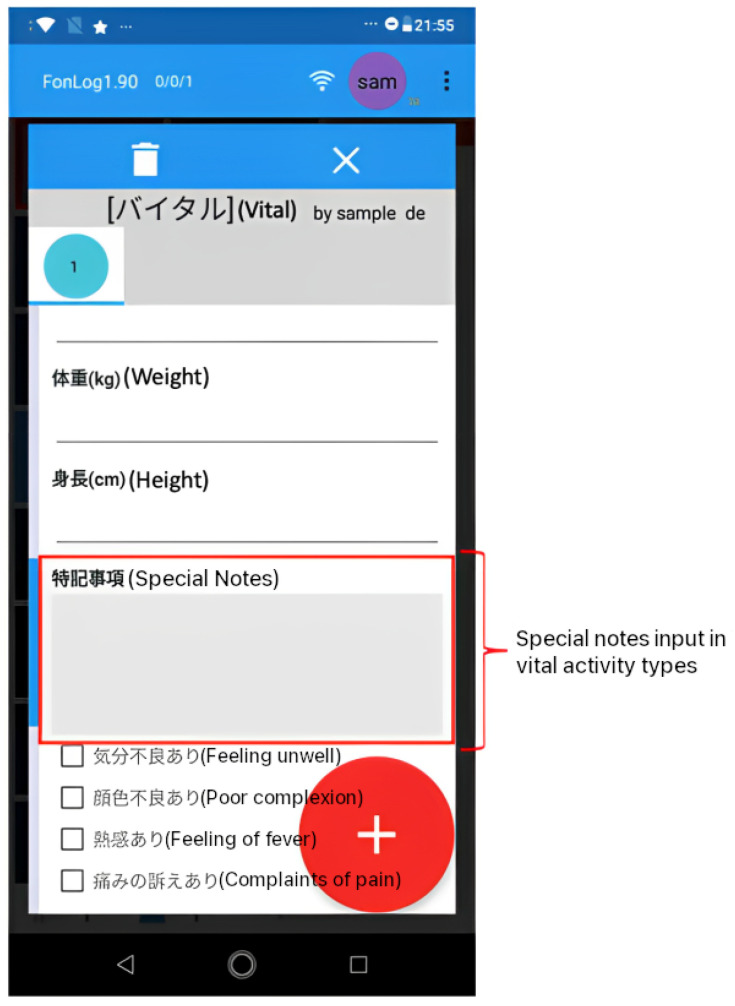
Special notes (特記事項) in the FonLog application.

FonLog’s special notes (特記事項) input is intended to capture more elderly information to allow caregivers to report specific patient conditions during activities. Caregivers can provide information in their own words through special notes, which provide a free-form input field. By providing caregivers with sentence suggestions to fill in the special notes, the caregiver’s task will undoubtedly be more efficient and effective regarding time and quality of records. Figure 1 shows the special notes display for the vital activity type, which functions to record extra information about the patient’s vital activity.

One of the inherent challenges in dealing with nursing care records lies in their diverse sentence structures. These records exhibit a rich collection of non-standard sentence constructions, making them inherently more complex than the standardized language often encountered in general texts [12]. This diversity arises from the varied nature of elderly histories, medical observations, and treatment plans, which can manifest in different linguistic forms. Traditional language models, designed to focus on conventional grammatical structures, may encounter difficulties in accurately interpreting and generating content that mirrors the intricate sentence structures in care records.

Furthermore, the specialized medical terminology in nursing care records introduces additional complexity. These documents incorporate highly specialized medical terminology, ranging from specific drug names to detailed descriptions of medical conditions and treatment procedures. The intricate vocabulary employed in healthcare documentation is crucial for precision and clarity. Still, it poses a considerable challenge for language models and evaluation metrics that may not be well-versed in the details of medical discourse. Consequently, assessing the similarity and relevance of sentences containing these specialized terms becomes a formidable task.

To effectively address the challenges posed by diverse sentence structures and specialized medical terminology in care records, we require an evaluation metric that mirrors the discernment of caregivers. This metric should possess the ability to assess the generated sentence suggestions for high information similarity with sentences in the care record database, serving as the ground truth. It is crucial that the evaluation metric not only evaluate grammatical accuracy but also recognize nuanced language structures and specialized medical terminology, aligning closely with the expertise of healthcare practitioners.

The current evaluation metrics, however, fall short of addressing these two challenges due to their limitations in recognizing diverse language structures and specialized medical terminology. Existing metrics struggle to navigate the complexity of varying sentence construction and evaluate language adequacy, thus diminishing their effectiveness in the context of nursing care notes. Additionally, their inability to interpret various pieces of medical terminology further underscores the need for more sophisticated evaluation approaches. Thus, developing metrics to address these limitations is critical to accurately assessing generated sentences, especially in the complex context of nursing care records.

### 2.2. Sentence Suggestion

Several research studies related to sentence suggestion have used keyword sentence completion. In existing research, rule-based, n-gram, and language models have been applied. Asnani et al. [4] explained that sentence completion utilizes techniques such as n-gram language models, neural network-based language models, and Markov chain methods. N-gram and Markov language models are easy to understand and apply for short and simple texts. They also discussed the advantages of neural network-based language models, which can model words over long distances but require a lot of data to train and are expensive and time-consuming. In another study, Mirowski and Vlachos [13] researched ways to improve the performance of recurrent neural network (RNN) language models by incorporating the syntactic dependencies of a sentence to have the effect of bringing in a context relevant to the word being predicted. In general, it can be concluded that this model is designed to learn word and grammar representations from text data and used to complete sentences automatically. The dependency recurrent neural language model (DRNLM) integrates word representation learning, grammar learning (dependency learning), and word order learning (recurrent learning) to produce accurate sentence representations. The authors evaluated DRNLM on three different datasets, namely the TREC dataset, MCScript dataset, and the CommonsenseQA dataset. In the evaluation, DRNLM outperformed state-of-the-art methods on all datasets. In their research, Irie et al. [14] investigated the use of recurrent neural networks (RNNs) and bi-directional LSTM-RNN (long short-term memory) variations in estimating sentence probabilities. The research included two experiments: first, they examined the effectiveness of using forward and backward RNNs in estimating sentence probabilities; secondly, they tested the combined methods of forward and backward RNNs, as well as bi-directional LSTM-RNNs, in estimating sentence probabilities. The results showed that using forward and backward RNNs separately resulted in relatively low accuracy in estimating sentence probabilities. However, when both methods were combined, the results were significantly better. In addition, the results of the bi-directional LSTM-RNN were better than those of the forward and backward RNNs separately. However, a bi-directional LSTM-RNN is more complex regarding neural network structure and computation time. Therefore, the authors concluded that combining forward, backward, and bi-directional LSTM-RNNs is the most effective method for estimating sentence probabilities. Rakib et al. [15] developed a Bangla word prediction model using a GRU (gated recurrent unit)-based recurrent neural network (RNN) and an n-gram language model. The aim of the research ws to improve word prediction accuracy and sentence completion in Bangla. The results show that the GRU model produced better accuracy in word prediction and sentence completion than the conventional RNN model. The research shows that combining an n-gram language model and a GRU model can significantly improve word prediction accuracy and sentence completion.

In the pursuit of determining the efficacy of the sentence suggestions produced by the model and their reflective application in care record sentences, using a robust evaluation metric is imperative. We identified noteworthy assessment variations through a comprehensive analysis of existing metrics and a subsequent comparative examination against human evaluations. This observation underscores the need for an evaluation metric that gauges the quality of sentence suggestions and aligns closely with expert opinions. The complexities of nursing care records demand a customized evaluation approach that goes beyond conventional metrics and reflects the caregiver’s evaluation. Thus, developing a specialized evaluation metric customized to the complexities of care records is crucial.

### 2.3. Current Evaluation Metrics

The selection of four current evaluation metrics, i.e., BERTScore, cosine similarity, BLEU, and ROUGE, is a deliberate choice based on several considerations. Firstly, these four metrics are widely recognized and extensively used in natural language processing. Their prevalence in the literature and adoption in various studies make them applicable benchmarks for comparison. Secondly, their diverse methodological approaches and shortcomings in evaluating sentences allow researchers to generate evaluation metrics suitable for nursing care records. BERTScore emphasizes contextual embeddings, cosine similarity assesses the vector space similarity, BLEU measures n-gram precision, and ROUGE gauges overlap in word sequences. This diversity allows for a comprehensive evaluation, considering various linguistic aspects. It is important to note that although other metrics are available, the selected evaluation metrics represent a balanced and representative subset, thereby ensuring a thorough assessment of both traditional and state-of-the-art metrics. Additionally, these metrics are considered industry standards, providing a solid basis for comparison and facilitating a comprehensive understanding of the proposed EmbedHDP. In Table 2 below, each current evaluation metric is described, along with its mechanism and limitations.

From Table 2, valuable insights can be gained for adaptation and improvement by examining the intricacies of the evaluation mechanisms of each method and understanding their limitations. Reviewing these metrics provides a solid foundation for identifying crucial points that can be utilized to refine and enhance the evaluation process. For instance, the strength of word embedding lies in its ability to recognize proximity between words based on their vector representations. On the other hand, the weakness of n-gram overlap is its tendency to ignore semantic coherence, failing to consider the context and meaning of words within a sentence.

Based on the related works discussed earlier, our research motivation is to propose an evaluation metric that aligns effectively with caregivers’ opinions. Recognizing the limitations of existing metrics in capturing the complexities of diverse sentence structures and specialized medical terminology, our objective is to develop a metric specifically relevant to the complexities of sentence suggestion generation in the context of nursing care records.

## 3. Proposed Method for Evaluation Metrics

We propose EmbedHDP, a new metric to address the challenges inherent in nursing care record sentences, such as varied sentence structures and medical terminology. Utilizing the hierarchical Dirichlet process (HDP) for training is practical because it provides flexibility. Since it does not limit the number of topics generated by the model, this approach is helpful for non-standard sentence structures where the resulting topic can be more optimal. Using HDP with a hierarchical approach can provide the ability to identify and organize topics in an orderly fashion. It means that HDP can capture more complex and in-depth structures in data by identifying main topics and more specific sub-topics or sub-sub-topics. Word embedding also plays an important role, specifically in mapping vector distances between the words in the nursing care records and then performing unsupervised training with a dictionary containing a collection of words in the nursing care notes.

The data utilized in our study were sourced from collaborative partnerships with various elderly facilities in Japan. It is important to note that we diligently ensured privacy protection by maintaining anonymity and confidentiality within the dataset. Our commitment to safeguarding sensitive information reflects our dedication to ethical data practices and compliance with privacy regulations. Through these established collaborations, we aim to contribute valuable insights that benefit our research and the overall well-being of the elderly community in Japan.

### 3.1. Hierarchical Dirichlet Process

The hierarchical Dirichlet process (HDP) is a topic modeling technique used to extract topics from sentences. In general, topic modeling is a method used to extract the primary topics from a large corpus of documents or text [20]. The essence of topic modeling is to identify hidden patterns in the text and discover interconnected topics based on words that frequently co-occur in documents. The HDP represents an enhancement of latent Dirichlet allocation (LDA), a method derived from the probability theorem [21] that aims to extract statistical structures of documents from various topics based on vocabulary distribution. HDP introduces a hierarchical structure that enhances its ability to capture latent topics within a corpus. Unlike LDA, HDP performs better in automatically determining the number of topics, eliminating users’ need to specify this parameter in advance. This adaptive capability makes HDP highly suitable for scenarios where the underlying topic structure is unknown. Here, we present the equation of an HDP model used to calculate the similarity between a generated sentence and its ground truth in the following Algorithm 1.
**Algorithm 1** Hierarchical Dirichlet Process Evaluation Model.**Input:** sentence1 = sentence similarity, sentence2 = ground truth **Output:** similarity score between sentence1 and sentence2 $S_{1}$ be the set of unique tokens in sentence1, $S_{2}$ be the set of unique tokens in sentence2, *D* be the dictionary formed by combining $S_{1}$ and $S_{2}$, $C_{1}$ be the Bag of Words (BoW) vector representing sentence1 in the corpus, $C_{2}$ be the BoW vector representing sentence2 in the corpus, $\mathrm{HDP}(D,\left[C_{1},C_{2}\right])$ be the trained Hierarchical Dirichlet Process model with dictionary *D* and corpus $\left[C_{1},C_{2}\right]$, $T_{1}$ be the topic distribution vector for sentence1 obtained from the trained HDP model, $T_{2}$ be the topic distribution vector for sentence2 obtained from the trained HDP model.

The similarity between sentence1 and sentence2 can be calculated using a similarity metric, for example:

$$\mathrm{Similarity}\left(T_{1},T_{2}\right)=\mathrm{cosine}\;\mathrm{similarity}\left(T_{1},T_{2}\right)$$

### 3.2. Word Embedding

Word embedding, a crucial component of natural language processing, has attracted considerable attention for its capacity to represent words in a continuous vector space. This computational technique, exemplified by models such as Word2Vec [22], GloVe [23], and FastText [24], transforms words into dense numerical vectors, capturing intricate semantic relationships and nuanced contextual information. Word embedding involves harnessing neural networks to learn from vast corpora, enabling the models to discern subtle linguistic patterns and relationships. These models create embeddings that encapsulate semantic similarities and syntactic structures by considering the co-occurrence of words in sentences. For instance, in sentiment analysis, word embedding allows algorithms to understand the sentiment behind words and phrases by recognizing their proximity in the vector space.

To examine the strengths and weaknesses of word embedding models, we can refer to Table 3 presented below.

Using word embeddings in natural language processing tasks offers several advantages. Unlike traditional one-hot encoding, word embedding provides a dense representation that preserves semantic information, facilitating more effective language understanding. For instance, the words “ナース (nurse)” and “介護者(caregiver)” might be located closer in the embedding space, reflecting their semantic similarity. Moreover, the ability of word embedding models to generalize well enhances their performance on unseen data, making them robust across diverse applications. In machine translation, for example, word embedding assists in capturing cross-language semantic relationships, improving translation accuracy for words with similar meanings but different linguistic expressions [25]. Additionally, word embedding enables more accurate matching of user queries with relevant documents in information retrieval by understanding the contextual similarities between words. As a result, word embedding stands as a crucial technique, advancing the capabilities of computational linguistics and bolstering the efficiency of diverse natural language processing applications.

Several studies have assessed the efficacy of various word embedding models spanning diverse linguistic contexts and applications. Investigations have ranged from comparing pre-trained word embedding vectors for word-level semantic text similarity in Turkish [26] to evaluating neural machine translation (NMT) for languages such as English and Hindi [27]. Additionally, the accuracy of three prominent word embedding models within the context of convolutional neural network (CNN) text classification [28] has been explored. The culmination of these studies suggests that the fastText word embedding model consistently outperforms its counterparts. The fastText model was selected as the optimal choice in the specific domain of our study, focusing on nursing care records consisting of Japanese sentences written by caregivers to report the progress and condition of the elderly. Its ability to handle infrequent or uncommon words by generating vectors for sub-words makes fastText exceptionally reliable in this scenario. The versatility and robustness exhibited by the fastText model underscore its effectiveness, making it a preferable choice in applications involving diverse and specialized vocabularies.

### 3.3. EmbedHDP

**Figure 2:**
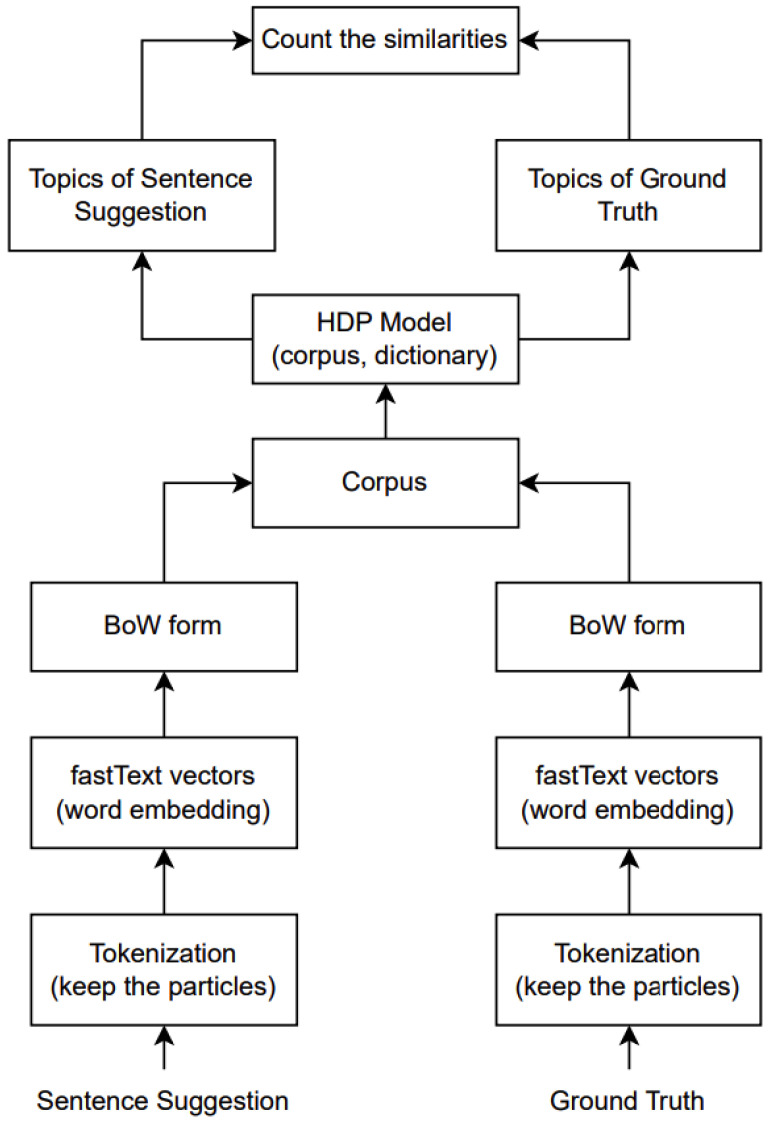
EmbedHDP architecture.

**Figure 3:**
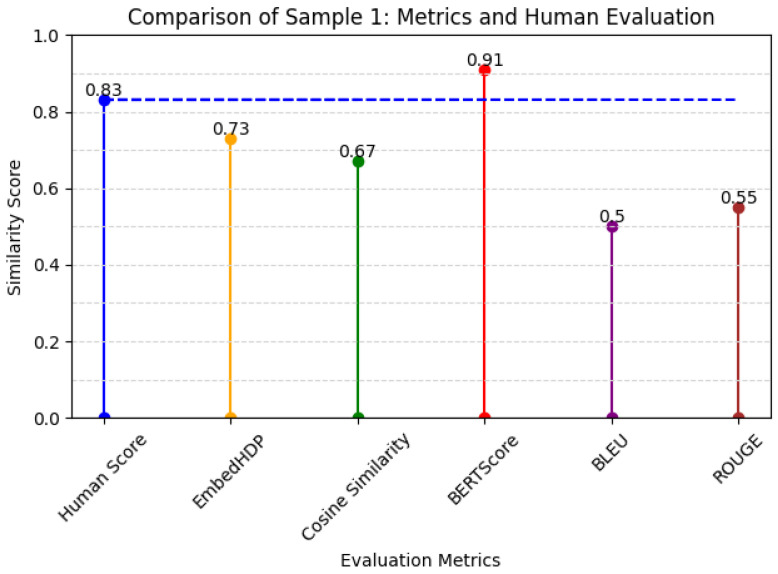
Metrics and human evaluation of sample 1.

**Figure 4:**
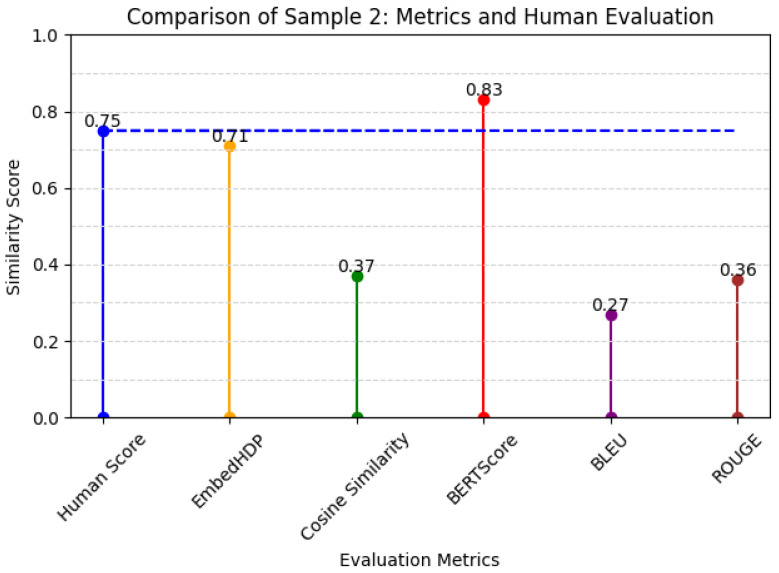
Metrics and human evaluation of sample 2.

Our original motivation was to address the specific characteristics of nursing care record sentences, which are often short and incomplete, displaying a prevalence of medical terminology. Thus, our proposed evaluation metric successfully achieved our intended motivation or goal. The method demonstrated enhanced accuracy compared to other evaluation metrics, signifying a notable advancement in the accurate evaluation of sentence suggestions within the unique context of nursing care records. Graphically, the architecture of our model can be visualized as shown in Figure 2. The EmbedHDP model has proven to be highly effective in handling these challenges inherent in nursing care record sentences, as shown in Figure 3 and Figure 4.

As illustrated in Figure 2, pre-processing sentences before being trained by HDP involves several key steps. First, the sentence is tokenized, a process whereby it is separated into individual tokens. We skip stemming and lemmatization at this stage while retaining particles attached to words. Once tokenized, these tokens are converted into vectors using fastText. Before processing them by the HDP model, we transform the vectors for each token into a BoW format. Afterwards, we merge the vectors from sentence suggestions with the ground truth, treating them collectively as the corpus. This comprehensive pre-processing workflow aims to prepare the data for optimal training and analysis by HDP, ensuring that the model is fed with comprehensively represented and formatted input. EmbedHDP’s pre-processing is explained in more detail as follows.

#### 3.3.1. Tokenization

The Japanese language has attracted attention with its unique linguistic structure, where verbs occupy the final position in the sentence [29]. In the tokenization process of Japanese, we utilize the Mecab library (-Owakati) [30]. We omit the lemmatizing and stemming processes. Additionally, the Japanese language contains unique particles to indicate subjects, objects, or other information. Another step in the tokenization process is preserving the particles attached to each word. Linguistic particles in Japanese refer to a distinctive feature of the Japanese language where small words or particles convey grammatical relationships and meaning in a sentence [8]. These particles play a crucial role in indicating the subject, object, direction, or emphasis of a statement, and their presence significantly influences the overall meaning of a sentence. The particles remain attached to words during the tokenization process, as shown in Table 4. This decision ensures that the additional information encapsulated in these particles remains intact, avoiding loss during the analysis process and enabling optimal utilization.

#### 3.3.2. Creating the Corpus

The corpus is the most crucial element in training HDP to derive topics. By default, corpus generation involves converting tokens within sentences using the BoW model. To achieve optimal results in HDP and facilitate the comparison of similarity between two sentences, the corpus is generated with the assistance of the fastText model. Specifically, we use cc.ja.300.bin, which encompasses a 7 GB vector in the Japanese language. An advantage of this model is its capability of generating vectors even for less familiar words, such as “介護者”, meaning caregiver. This attribute enhances the model’s versatility and ensures comprehensive coverage in vector representation.

The additional challenge is that the HDP model exclusively accepts the BoW format. This implies a direct processing barrier for vectors generated by fastText into the HDP model. The subsequent steps to overcome this hurdle involve converting the vectors into BoW format with the following stipulations:

1. Set vector length: Assign a fixed vector length in the BoW format, specifically 10. This decision is based on the consideration that our sentences are not excessively long, thereby mitigating potential biases arising from vector length discrepancies.
2. Highest-frequency elements: Select elements based on their highest frequencies, assuming the highest frequency serves as a representative token for each element.
3. Scaling vector: Due to the considerable length of vectors produced by fastText, the resultant BoW-formatted vectors are exceedingly small (0.000×). This phenomenon leads to nearly identical topics when trained on the HDP model. To counteract this issue, each vector is multiplied by 100, ensuring positive values throughout the vector and resolving the disparity.
4. BoW Representation: The outcome of these steps is the acquisition of BoW-formatted vector representations for each token in both sentences. This transformation facilitates seamless compatibility with the HDP model during the training process.

#### 3.3.3. Dictionary

The hierarchical Dirichlet process (HDP) is a statistical model addressing resource allocation challenges in data clustering. In the context of HDP, the term “dictionary” refers to a stochastic distribution concept employed to represent the distribution of topics within a dataset. Specifically, within the EmbedHDP framework, the dictionary encapsulates the local topic distribution specific to the nursing care record dataset. The dictionary fundamentally plays a role in determining the extent of the topics present across the entire dataset and the proportion of topics applicable to specific data clusters.

In this study, we comprehensively compiled a set of 268 unique words that profoundly represent nursing care record applications. These words, such as リドメックスローション (redomex lotion), 病院(hospital), リハビリテーション (rehabilitation), 不安(anxiety), 感染(infection), and others, were strategically chosen to represent diverse aspects of nursing care records. Incorporating such a dictionary in the model contributes to a nuanced understanding of the topics prevalent within nursing care record applications, fostering insights into the unique linguistic characteristics inherent in this domain.

In line with the explanation above, below, we present an example of a sentence suggestion compared with the ground truth. From this example, we can see that the hierarchy in EmbedHDP provides flexibility because it does not require prior specification of the number of groups or topics available. This is an important point in non-standard sentence structures. Hierarchies provide flexibility and adaptability, making them an effective tool for modeling data with complex and uncertain structures. Below, we provide an example of using EmbedHDP that produces useful judgments in incomplete sentences.

In Table 5, we present the respective scores assigned by humans to the benchmark, EmbedHDP, and several other current evaluation metrics.

As demonstrated by the examples described in Table 5 and Figure 3, EmbedHDP outperforms other evaluation metrics in coefficient scores with short example sentences with no subjects. This proves that the recognition of the information using the topic approach developed by EmbedHDP follows a human evaluation approach.

Another challenge in care records involves the presence of medical terminology within sentences. EmbedHDP can address this challenge using a dictionary containing words relevant to care records. The dictionary is utilized during model training to compute potential topic distributions for each word within the sentences. In addition, word embedding models play a critical role because their vector-representational strength enables the capture of semantic meaning in individual words. Essentially, the strength of these vector representations lies in their ability to bring vectors of words with similar meanings closer together in vector space. Below, we provide an example of how word embedding helps provide a significant assessment based on expert opinion. The example involves a sample of data derived from word embedding-based scoring optimization.

Below, we present human scores as a benchmark compared to EmbedHDP and several other current evaluation metrics for Sample 2 in Table 6.

From the above example, we can conclude that EmbedHDP is able to provide a relevant evaluation resembling human evaluations.

However, the EmbedHDP model may not be as effective when dealing with relatively long or complex sentences that contain more than 14 words. This limitation may arise from the substantial amount of information within lengthy sentences, making it challenging to capture semantic nuances comprehensively across the entire sentence.

**Figure 5:**
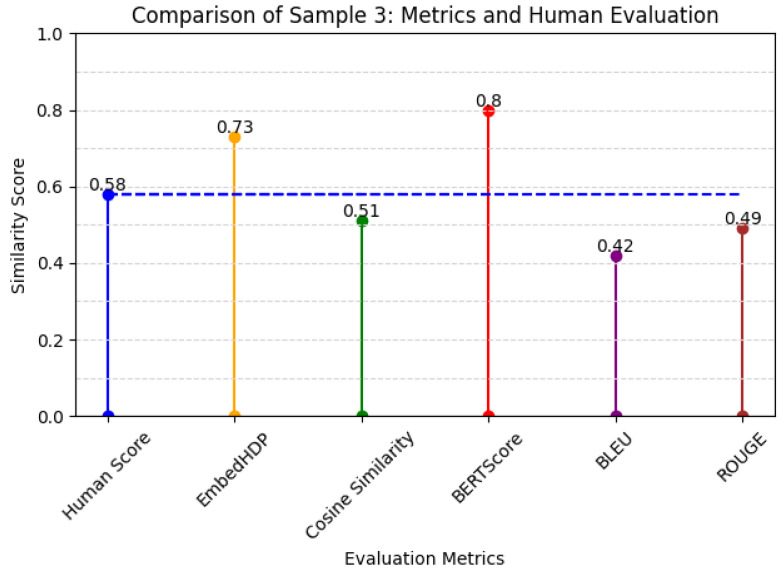
Metrics and human opinion assessment of Sample 3.

Figure 5 reports the respective scores assigned by humans to the benchmark, EmbedHDP, and several other current evaluation metrics for Sample 3 in Table 7.

## 4. Evaluation

The objective of EmbedHDP is to enhance the performance of evaluation metrics when assessing sentence suggestions generated by the model in comparison with their respective ground truths within the scope of care records. The evidence presented in this section demonstrates that EmbedHDP consistently outperforms current evaluation metrics in evaluating 228 data samples, as indicated by the higher correlation coefficient score. This indicates that EmbedHDP consistently produces similarity scores between sentence suggestions and ground truths that closely align with the scores provided by human evaluation. In other words, EmbedHDP accurately measures the similarity between the two sentences by capturing underlying topics.

### 4.1. Filtering the Data Sample

In Section 3, we discussed the limitations of EmbedHDP when handling relatively long sentences. Due to the potential challenge posed by longer sentences, which may contain more intricate information, it can be challenging for a model to capture the comprehensive semantic meaning of the entire sentence effectively. To address this, a data-filtering criterion was implemented to test the proposed evaluation model. Specifically, we decided that only sentences with 13 words or fewer would be used in the testing phase. This limitation was put in place to ensure that the evaluation model was assessed under conditions where sentences were kept relatively concise. By focusing on shorter sentences, the model can more accurately evaluate its ability to understand and generate context with optimal relevance and precision within a constrained linguistic scope. The filtration process can be automated with the following pseudo-code: Input: sentence1 (sentence suggestion), sentence2 (ground truth)

 if len(sentence1) > 13 or len(sentence2) > 13:

return “Both sentences eliminated”.

Based on the aforementioned conditions, the initial dataset, which originally comprised 390 data points, was reduced to 320 data points due to the imposed criteria. Additionally, 70 data points were identified as outliers and subsequently excluded from the dataset. In statistical analysis, identifying and handling outliers is a common practice to ensure the robustness and reliability of the data. Outliers, which are data points significantly different from the majority of the dataset, can substantially impact statistical measures. By excluding these outliers based on the specified criteria, the dataset was refined to a more representative and manageable size, consisting of 320 data entries. This process contributes to more accurate analysis and interpretation of the dataset, aligning with best practices in data pre-processing.

### 4.2. Utilized Evaluation Metrics

In the comprehensive exposition above, we conveyed that EmbedHDP is a potential solution for evaluating models applied to care record sentences, addressing two primary challenges. Furthermore, we substantiated that EmbedHDP successfully yielded assessments that align more closely with expert opinions than other evaluation metrics.

The challenges in evaluating care record sentences, such as diverse sentence structures and specialized medical terminology, require a model that can effectively discern nuances. EmbedHDP, through its incorporation of hierarchical Dirichlet processes and domain-specific dictionaries, demonstrates the ability to navigate these intricacies.

Three important variables serve as the basis of EmbedHDP, contributing to its robust functionality in evaluating sentence suggestions in nursing care records. First, the hierarchical approach establishes the framework for handling data clustering within a hierarchical structure, offering adaptability to the inherent complexity and uncertainty present in the data. This hierarchical model facilitates the representation of nuanced relationships between topics, thereby enhancing the model’s capacity to capture intricate patterns within the data.

Secondly, word embeddings are key to generating vectors with expansive semantic values. The embedding matrix systematically maps words or tokens to continuous vectors, fostering a distributed representation that encapsulates subtle semantic relationships between words. This approach enhances the model’s ability to grasp contextual nuances, contributing to the evaluation of coherent and contextually relevant sentence suggestions.

Lastly, combining a dictionary with the Dirichlet distribution plays a pivotal role in determining the existence and prevalence of topics within each document. The dictionary provides a repository of words systematically paired with the Dirichlet distribution, guiding the allocation of topics and influencing the overall thematic composition of the generated sentences. This synergistic interplay of hierarchical structures, word embeddings, and dictionary-based topic determination collectively forms the foundation of EmbedHDP, ensuring its adaptability, semantic richness, and effectiveness in generating contextually relevant sentence suggestions. Table 8 below shows that EmbedHDP outperforms current evaluation metrics in coefficient score on 320 test data points.

EmbedHDP surpassed current evaluation metrics when assessing the quality of sentence suggestion generation against the corresponding ground truth. Employing coefficient correlation parameters in human evaluation, EmbedHDP outperforms other evaluation methods, with a score of 61%, followed by cosine similarity at 59% and BERTScore at 58%. This substantiates the effectiveness of EmbedHDP as the proposed primary evaluation metric for the assessment of sentence suggestion generation in care records. Notably, the observed higher linear relationship between EmbedHDP and human scores compared to other evaluation models underscores its robust performance in capturing the nuances of human expert opinions.

The 70 identified outliers can be classified as one of the limitations in both EmbedHDP and other evaluation metrics. The presence of outliers can pose a significant challenge in data evaluation and analysis, including within the context of utilizing EmbedHDP and current evaluation metrics. Outliers can impact evaluation results significantly, particularly if the model or metric is not designed to handle extreme variability. In the context of EmbedHDP, identifying and addressing outliers may become a focus of future development to enhance the model’s robustness against unusual data variations. Table 9 shows the correlation coefficients for 70 data outliers.

### 4.3. Benchmarking Method

In the preceding section, we delved into the mechanisms and limitations inherent in current evaluation metrics. Additionally, we explored the challenges posed by care record sentences and elucidated how our proposed evaluation model, EmbedHDP, is poised to address these challenges. Examining current evaluation metrics provided insights into their operational mechanisms and constraints. This understanding sets the stage for the introduction and justification our proposed model, EmbedHDP, which offers a novel approach to evaluating sentence suggestions within the context of care record sentences. By acknowledging and addressing the specific challenges posed by the diverse sentence structures and specialized medical terminology in care records, EmbedHDP aims to provide a more nuanced and contextually relevant evaluation. The following example illustrate how EmbedHDP can address some of the limitations inherent in current evaluation metrics when facing the challenges posed by nursing care records.

**Figure 6:**
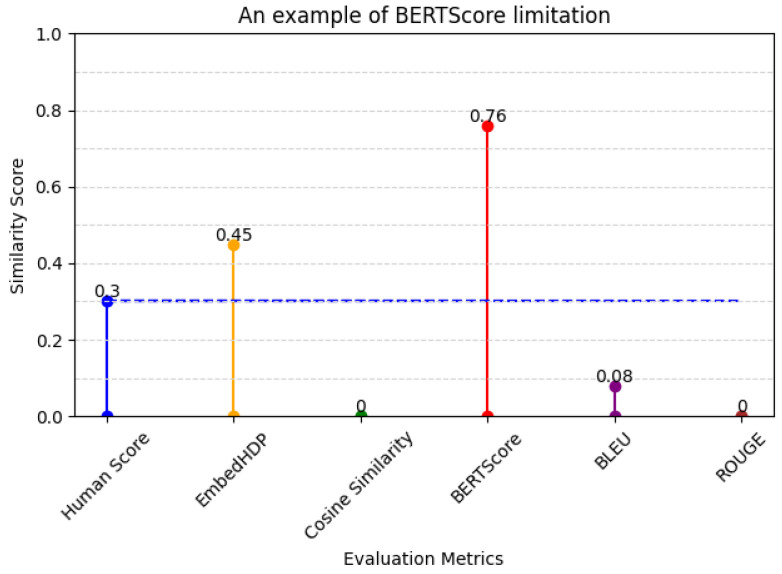
Metrics and human evaluation assessment of BERTScore’s limitation sentence.

1. The example of BERTScore’s limitations when evaluating short sentences (diverse sentence structures) and those containing medical information (specialized medical terminology) in Table 10 and its evaluation score in Figure 6.

**Table 10:** An example of BERTScore’s limitations.

Sentence Suggestion	Ground Truth
頻繁な少量の排尿。 (frequent small amount of urination)	排便中量あり。 (There was a large amount during defecation).

**Figure 7:**
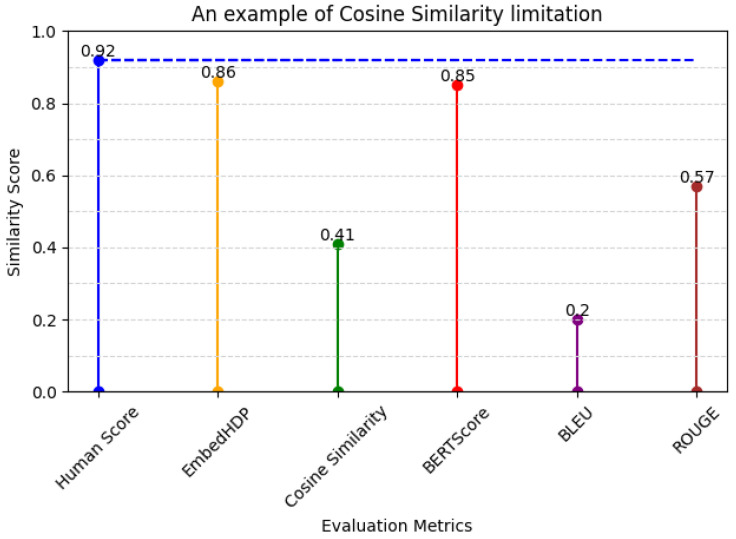
Metrics and human evaluation assessment of cosine similarity’s limitation sentence.

2. The example of cosine similarity’s limitations when evaluating short sentences (diverse sentence structures) and those containing medical information (specialized medical terminology) in Table 11 and its evaluation score in Figure 7.

**Table 11:** An example of cosine similarity’s limitations.

Sentence Suggestion	Ground Truth
吐き気あり報告入れる。 (report nurse)	吐き気訴えあり。 (complaints of nurse)

**Figure 8:**
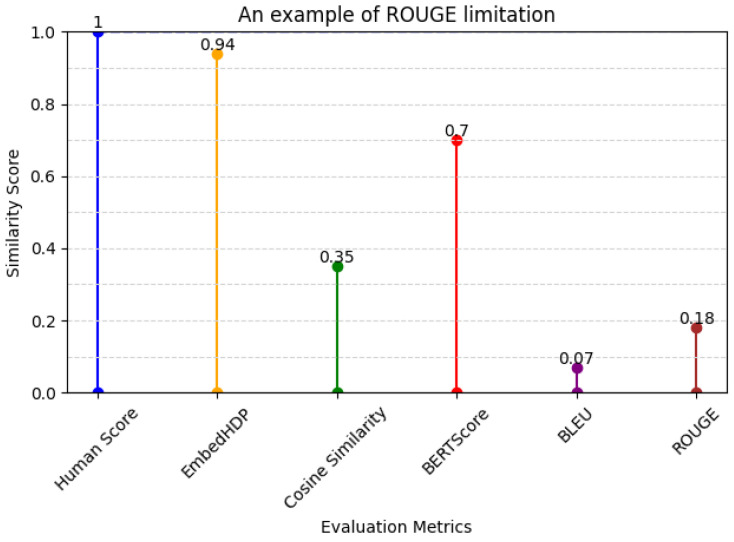
Metrics and human evaluation assessment of ROUGE’s limitation sentence.

3. The example of ROUGE’s limitations when evaluating different structures of sentences (diverse sentence structures) in Table 12 and its evaluation score in Figure 8.

**Table 12:** An example of ROUGE’s limitations.

Sentence Suggestion	Ground Truth
気分訴えなし (no mood complaints)	気分不良はないと本人言われる (he says he doesn’t feel unwell)

**Figure 9:**
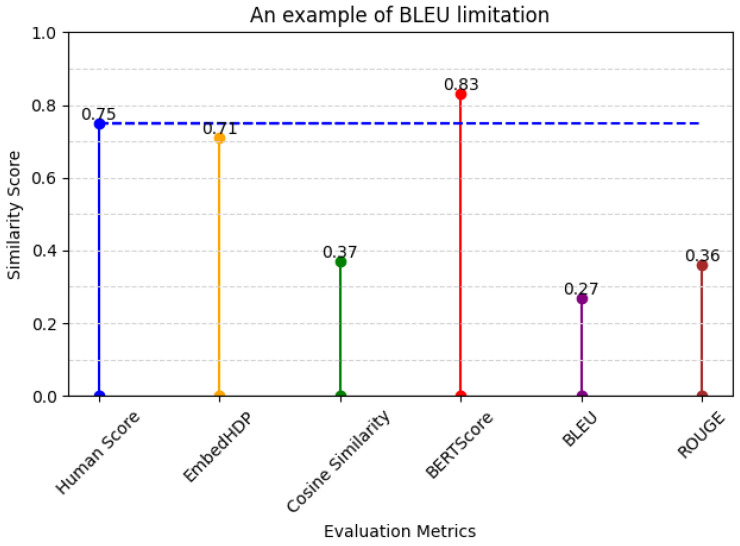
Metrics and human evaluation assessment of BLEU’s limitation sentence.

4. The example of BLEU’s limitations when evaluating different structures of sentences (diverse sentence structures) and those containing medical information (specialized medical terminology) in Table 13 and its evaluation score in Figure 9.

**Table 13:** An example of BLEU’s limitations.

Sentence Suggestion	Ground Truth
熱があったので、看護師に報告して中止しました。 (I had a fever, so I informed the nurse and cancelled the session)	熱発の為、ナースに報告し中止。 (Due to fever, the nurse was informed and the procedure was discontinued)

## 5. Discussion

The aim of this research was to construct more effective evaluation metrics that capture semantic information within advisory sentences found in healthcare records and to generate assessments that align closely with human scores. These evaluation metrics can be applied to evaluate sentence suggestions in nursing care records without the necessity of human inclusion. However, it is essential to acknowledge that EmbedHDP still has limitations, which require careful analysis for future development. One significant challenge is EmbedHDP’s vulnerability when analyzing the similarity between two sentences in nursing care records, especially when the sentences contain 14 or more words. Despite these limitations, this research underscores the potential of EmbedHDP as a promising tool in advancing the accuracy of automated evaluations in nursing care records. This research offers valuable insights for both current applications and future refinements.

In Section 4.1, we discussed the crucial aspect of data filtration undertaken for this study. This strategic decision was derived from the inherent limitation of our proposed EmbedHDP model in evaluating sentences comprising 14 words or more. The reason behind this choice is grounded in the observation that longer sentences tend to be more complex and contain overlapping information. Consequently, EmbedHDP requires refinement to effectively address the challenges of longer sentences. Acknowledging this limitation underscores the commitment to transparency in our research. This paves the way for future enhancements and optimizations to improve EmbedHDP’s performance in handling intricate and lengthy sentence structures.

Subsequently, as outlined in Table 1, the FonLog care record application contains 31 activity types that require sentence suggestions. However, for our current study, we only used the dataset associated with the vital (バイタル) activity type. While this subset is considered representative of the overall structure of nursing care record sentences, it is acknowledged that additional conditions or contexts within other activity types could further enrich EmbedHDP’s training. Future endeavors may explore incorporating datasets from diverse activity types to enhance the model’s robustness and versatility. This approach could provide a more comprehensive and nuanced understanding of the linguistic patterns across various care-related activities in the nursing care application.

Technically, in developing sentence suggestion systems for elderly care records, it is important to consider the precision of evaluation metrics. Current metrics may not fully reflect the ability to analyze the quality of suggested sentences, which is crucial for their application within nursing care record systems. This significance stems from the generated sentence suggestions being closely linked to critical information about the elderly, and any inaccuracies could pose potential risks. After reviewing several selected evaluation metrics, we proved that enhancement has the potential to make them more comparable to human evaluation standards. Enhancing these metrics is essential to ensure the accuracy and reliability of the suggested sentences within nursing care record applications, particularly in the context of elderly health information.

Another potential approach for enhancing the evaluation of sentence suggestions in care record applications involves fine tuning existing metrics, particularly focusing on well-established evaluation models like BERTScore. This strategic approach acknowledges the necessity of domain-specific evaluations that can accurately capture the intricacies of language within care records. While fine tuning requires a significant time investment, especially for tasks such as generating contextual embeddings specific to the care record domain, the potential benefits are substantial. The resulting fine-tuned metrics have the potential to provide a more nuanced and accurate assessment of the generated sentence, contributing to the continual refinement of natural language processing techniques tailored to the intricacies of healthcare-related text. In care records, where language is highly specialized and context-dependent, adapting evaluation metrics like BERTScore through fine tuning is a strategic move.

The applicability of EmbedHDP to domains besides nursing care records requires careful consideration, primarily in evaluating its adaptability to different data characteristics. EmbedHDP has effectively evaluated sentences with short and seemingly incomplete structures, where sentences may lack subjects or objects. This means that it can be adapted to domains with similar characteristics. The model’s ability to handle incomplete sentences aligns with scenarios where linguistic structures may vary.

Accordingly, the relevance of the task should be reviewed, as EmbedHDP was initially designed to assess sentence suggestions within nursing care records. Specific domain-related words in nursing care records are acceptable for the model’s performance, particularly when evaluating the similarity between sentence suggestions and the ground truth within the scope of nursing care records. The incorporation of word embeddings and dictionaries proves instrumental in capturing the nuances of domain-specific language. One key consideration is the involvement of domain experts, who can contribute invaluable insights to enhance the model’s relevance and effectiveness. Therefore, before extending the application of EmbedHDP to different domains, a thorough understanding of data characteristics, task relevance, and expert involvement is essential for successful adaptation.

## 6. Conclusions

In this paper, we aimed to provide evaluation metrics designed to assess the quality of sentence suggestions in nursing care record applications, specifically tailored to record information about the elderly. Nursing care records present specific challenges, including different sentence structures and specialized medical terminology. We introduced evaluation metrics that address the two main challenges in care records by employing a methodology that computes topic similarity using word embedding vectors. This innovative approach aims to overcome the challenges of diverse sentence structures and specialized medical terminology in care records. By leveraging the power of word embedding vectors, our proposed evaluation metrics strive to capture the semantic nuances and context-specific information inherent in healthcare-related texts, providing a more accurate and contextually relevant assessment of sentence suggestions in nursing care records. This approach reflects a commitment to advancing evaluation techniques tailored to the unique linguistic characteristics of healthcare data.

Furthermore, this study significantly contributes to nursing care applications through the application of a comprehensive approach. The systematic collection of a dataset comprising 390 pairs of sentences diligently evaluated by three caregivers provided a reliable benchmark ground truth for assessing the quality of sentence suggestions. The testing and analysis of current evaluation metrics, such as BERTScore, cosine similarity, ROUGE, and BLEU, yielded valuable insights into their effectiveness in nursing care records. Additionally, by introducing an innovative evaluation model characterized by a novel approach, EmbedHDP demonstrated considerable performance improvement compared to existing models. This enhancement ensures a closer alignment with caregiver assessments, marking a substantial step forward in advancing the accuracy and reliability of sentence suggestion evaluations within the realm of nursing care applications.

Future works will leverage established models such as BERTScore and fine tune their contextual embeddings to align with the specific characteristics of care record sentences. A comparison with EmbedHDP will provide insights into the strengths and weaknesses of each model. Additionally, efforts will be directed towards refining both evaluation models by gaining a deeper understanding of their mechanisms. This iterative process of refinement and comparison contributes to the continuous improvement and adaptation of evaluation techniques for sentence suggestions in the care records domain. 

## Figures and Tables

**Table 1:** Special notes of input according to activity type.

Activity Type	Record Type
1. バイタル (vitals)	
2. リハビリ・レク (rehabilitation/recreation)	
3. 往診・受診 (house calls/visits)	
4. 処置 (treatment)	
5. 入浴・清拭 (bathing/cleaning)	
6. 外出対応 (going out)	
7. 活力朝礼・ラジオ体操 (vitality morning/radio exercise)	
8. 特記事項・連絡事項 (special notes/notifications)	
9. 送迎 (transportation)	
10. 事故等緊急対応 (emergency response)	
11. 就寝前食事 (meal before bedtime)	
12. モーニングケア (morning care)	1. 特記事項 (special notes)
13. ナイトケア (night care)	2. 状態・特記事項 (condition/special notes)
14. その他食事(other meals)	3. 連絡事項 (notifications)
15. 家族・来客対応 (family/visitor support)	4. 傷の状態・特記事項 (status/special notes)
16. 家族・医師連絡 (family/doctor contact)	
17. 手書き記録 (handwritten records)	
18. 入院 (hospitalization)	
19. 離床・臥床介助 (assistance with getting out of bed and lying down)	
20. 食事・服薬 (meals and medication)	
21. おやつ (snacks)	
22. 更衣介助 (assistance with changing clothes)	
23. 口腔ケア (oral care)	
24. 排泄 (excretion)	
25. 日中利用者対応 (support for daytime user)	
26. 夜間利用者対応 (support for nighttime user)	
27. 朝食 (breakfast)	
28. 昼食 (lunch)	
29. 夕食 (dinner)	
30. 洗面介助 (washing assistance)	
31. 外泊 (overnight stay)	

**Table 2:** Comparison of evaluation metrics for sentence suggestion in care records.

Evaluation Metric	Mechanism	Limitation
BERTScore [16]	Compares contextual embedding of reference and candidate sentences using pre-trained BERT models	It relies on pre-trained BERT models, which may not capture domain-specific nuances effectively
Cosine Similarity [17]	Calculates the cosine angle between two vectors to determine their similarity. It is frequently used to compare text documents in vector space.	Fails to account for word order and mistakenly rates semantic difference with similar word sets.
ROUGE [18]	Measures the overlap of n-grams and the longest matching sequence between a generated summary and reference texts.	Might overlook semantic accuracy, as it is based on lexical overlap, not considering the context or meaning of the words.
BLEU [19]	Scores machine translations by matching n-grams to reference texts and adjusting for translation length.	Can miss the adequacy and fluency of translation, as it primarily relies on n-gram overlap, ignoring semantic coherence.

**Table 3:** Strengths and weaknesses of word embedding models.

Word Embedding Model	Strengths	Weaknesses
Word2Vec	Semantic similarity and efficient contextual understanding	Out-of-vocabulary words, limited word level, and insensitive to word order
GloVe	Global context, effective for common words, and linear structure	Less effective for rare words and limited contextual understanding
fasText	Sub-word information, better representation for rare words, and efficiency	Computationally more demanding, requiring more memory usage

**Table 4:** Functions of several particles and verbs in Japanese.

Japanese Particle	Explanation and Example
は (wa)	The topic particle that indicates the topic or subject of a sentence. For example, “わたし は がくせい です” (Watashi wa gakusei desu) means “I am a student”.
へ (e)	Indicates direction or destination. For instance, “ともだち へ いきます” (Tomodachi e ikimasu) means “I am going to a friend”.
で (de)	Indicates the place or method by which which an action takes place. For example, “レストラン で たべます” (Resutoran de tabemasu) means “I eat at the restaurant”.
を (wo)	The object particle that indicates the object of an action. For example, “りんご を たべます” (Ringo o tabemasu) means “I eat an apple”.
の (no)	The possessive particle or connector between two nouns. For example, “わたし の くるま” (Watashi no kuruma) means “My car”.
ある (aru)	A verb indicating existence or possession. For example, “ほん が あります” (Hon ga arimasu) means “There is a book”.
あり (ari)	The past or formal form of the verb “ある” (aru), indicating existence.
する (suru)	A common verb meaning “to do”. For example, “しゅくだい を する” (Shukudai o suru) means “To do homework”.
なる (naru)	A verb meaning “to become”. For example, “せんせい に なりたい” (Sensei ni naritai) means “I want to become a teacher”.
し (shi)	A conjunction used to express two related actions or qualities. For example, “りんご し いちご” (Ringo shi ichigo) means “Apples and strawberries”.
て (te)	The ’te’ form of a verb, indicating an ongoing action. For example, “たべ て います” (Tabete imasu) means “I am eating”.
ます (masu)	A polite form of verbs indicating present actions. For example, “たべ ます” (Tabemasu) means “I eat” or “I will eat”.

**Table 5:** Sample 1 illustrates how HDP can effectively address incomplete or fragmented sentences.

Sentence Suggestion	Ground Truth
コルセット作ることを報告する (report making a corset)	コルセットを作ることを勧められる (advised making a corset)

**Table 6:** Sample 2 illustrates how word embedding can effectively address the similarity of words in both sentences.

Sentence Suggestion	Ground Truth
熱があったので、看護師に報告して中止しました。 (I had a fever, so I informed the nurse and cancelled the session.)	熱発の為、ナースに報告し中止。 (Due to fever, we informed the nurse and discontinued the treatment.)

**Table 7:** Sample 3 illustrates how sentence length affects the evaluation quality of EmbedHDP.

Sentence Suggestion	Ground Truth
両眼内障であること、右眼は緑内障疑いで眼圧が高くなっては弱い痛み止めを屯用で出しておくので飲んで心臓の状態が良いとの連絡あり (I was informed that I have bilateral eye disorders, that my right eye is suspected of having glaucoma, and that my intraocular pressure is high, so they give me a weak painkiller to take, and that my heart is in good condition.)	両眼白内障であること、右眼は緑内障疑いで眼圧が高くなっていること、だから目が見えにくくなっている、と説明を受けられ、眼圧を下げる点眼薬を処方されたこと (He explained to me that he had cataracts in both eyes, that his right eye had high intraocular pressure due to suspected glaucoma, and that he was having difficulty seeing and was prescribed eye drops to lower the intraocular pressure.)

**Table 8:** Comparison of EmbedHDP with other current evaluation metrics in 320 data.

Evaluation Metric	Correlation Coefficient
EmbedHDP	0.61
BERTScore	0.58
ROUGE	0.57
Cosine similarity	0.59
BLEU	0.53

**Table 9:** Comparison of EmbedHDP with other current evaluation metrics in 70 data outliers.

Evaluation Metric	Correlation Coefficient
EmbedHDP	0.25
BERTScore	0.35
ROUGE	0.34
Cosine similarity	0.35
BLEU	0.34

## Data Availability

The data presented in this study are available upon request from the corresponding author. The public dataset is available online at https://github.com/atoxcode/ss-in-ncr (accessed on 15 December 2023).

## Abbreviations

The following abbreviations are used in this manuscript:

BERT	Bidirectional encoder representations from transformers
ROUGE	Recall-oriented understudy for gisting evaluation
BLEU	Bilingual evaluation understudy
HDP	Hierarchical Dirichlet process
BoW	Bag of words
RNN	Recurrent neural network
DRNLM	Dependency recurrent neural language mode
TREC	Text retrieval conference
LSTM	Long short-term memory
GRU	Gated recurrent unit
GloVe	Global vector
NMT	Neural machine translation
CNN	Convolutional neural network
